# Notes and News

**Published:** 1888-07-21

**Authors:** 


### Notes and News.

Collected and Communicated.

A handsome presentation was made to Dr. Spence Watson at Newcastle on Monday, on the occasion of his silver wedding.

A total of £5,563 16*s*. has been remitted by the Lord Mayor to his Excellency the British Ambassador at Berlin, as the proceeds of the fund opened at the Mansion House on behalf of the sufferers by the floods in Germany.

Messrs. Baring and Messrs. Rothschild have each promised £1,000 towards the fund now being raised for the establishment of polytechnic institutes in South London. It is said that the fund already amounts to a very considerable sum, and that a list of subscriptions will shortly be published.

Mr. Ernest Renshaw, after a somewhat one-sided contest, beat Mr. H. J. Lawford, the champion tennis player, and now holds the championship. Mr. W. Renshaw, who held the honours for several seasons against all comers, is brother to the new champion.

General Boulanger is better. It is not added that he is wiser. Is it part of the order of creation that Frenchmen are to have only one period of childhood, and that that is to continue throughout life?

An eye department has just been opened at the Derbyshire General Infirmary, and Mr. E. Collier Green, a former house surgeon, was last Monday unanimously elected as the first ophthalmic surgeon. Mr. Green has always devoted special attention to ophthalmology. He filled the posts of clinical assistant at the Royal London Ophthalmic Hospital, and ophthalmic assistant at the Children's Hospital, Great Ormond Street. The directors of the Derbyshire Infirmary may be congratulated on their choice of a surgeon for the new department who both knows and loves his work.

On Tuesday, the 10th, T.R.H. Princess Beatrice and Prince Henry of Battenberg visited the Miller Hospital, Greenwich. The pretty hospital was decorated for the occasion, and Mr. Bristow (hon. sec.) read the address, in which Her Royal Highness was asked to name the female ward "The Princess Beatrice Ward." The address, which had been beautifully illuminated, was then handed to the Princess, who consented to the ward being named after her. The matron (Miss Edwards) was then presented to the Princess, whom she conducted round the wards, Her Royal Highness speaking a kindly word to each patient. A large number of visitors subsequently went through the hospital while a band discoursed pretty and lively music. Altogether the 10th was quite a jubilee day for both nurses and patients at the Miller Hospital.

Their Royal Highnesses the Prince and Princess of Wales opened the new buildings of the Great Northern Central Hospital on Tuesday. The North of London was *en fete* for the occasion. For a distance of nearly three miles north-west of the Angel the principal streets were gaily decorated, some of the larger shops being resplendent with plumes, flags, streamers, and other brilliantly-coloured bunting. Tens of thousands of people lined the route and cheered enthusiastically as the procession passed through the streets. Mr. C. T. Murdoch, M.P., welcomed the Prince and Princess on behalf of the Hospital Committee, and Mr. F. Dewey, the vestry clerk of Islington, read an address as representative of the parish authorities. The Prince said it gave the Princess and himself sincere pleasure to know that the new hospital was regarded by the North of London as its most fitting memorial of the Queen's Jubilee.

It was announced that the Princess of Wales would receive purses of not less than five guineas each at the opening ceremony of the new hospital buildings in North London. Large numbers of purse-bearers, principally little girls, were prepared with purses, and presented them to the Princess. The presentations were watched with great interest by a vast crowd, consisting largely of ladies, many of whom were evidently very near akin to the little ones. It was amusing to see how gracefully some of the children performed the ceremony of offering the purse, whilst others as evidently regarded the affair with much less gratification, some even passing by with head erect, as if they scorned to be ceremonious even on so ceremonious an occasion. The Royal pair were frequently seen to smile as the different varieties of juvenile court behaviour were presented to their view. The total amount realised by the purses was £1,050.

The proceedings at the opening ceremony of the Great Northern Central Hospital commenced in the finest of summer weather and closed in a perfect downpour of rain. The large marquee, which was of substantial construction, soon began to let in the flood. The torrents continued, and presently huge bags of water appeared at different portions of the roof, threatening to burst the canvas and deluge the unfortunate people. One adventurous gentleman, more bold than wise, essayed to push up one of the largest of the watery bulgings with the point of his umbrella. His temerity was speedily punished; the unlucky canvas burst, and a perfect deluge poured down upon his hatless head, drenching him from top to toe, amid roars of laughter from the amused audience. The rain continued for more than an hour; but everyone seemed in excellent spirits, and the proceedings of the day were brought to a close amid general congratulations.

Last Saturday the fifteenth annual collection on behalf of the Hospital Saturday Fund was taken in the metropolis. The collecting stations occupied numbered more than two thousand. The ladies who had undertaken the duty of attending at the stations were at their posts early in the morning, and displayed great assiduity and courtesy throughout a long and wearying day. The cab trade, as on previous occasions, entered into the subject with much heartiness. The men took charge of five hundred special boxes, and advertised the fund in all directions by adorning their whips with pennants and filling their windows with posters. The docks, railway companies, and other industrial centres rivalled each other in their efforts to make the day the most successful of its kind. By favour of Mr. Edgar Shand, twenty-five boxes had been placed on the river boats. A collection was made for the first time at the Tower of London, by permission of Lieut.-Colonel Milman. The workshop collection will be continued weekly until September 1st. It is hoped and expected that the fund will show a large increase on the amounts received in past years.

THE following vacancies are announced this week, the amount of salary in each case being given after the name of the institution :—

**Resident Medical Officers.**

Birmingham General Hospital. Assistant House Surgeon.—Board ; no salary.
Central London Ophthalmic Hospital, Gray's Inn Road. House Surgeon.—Board ; no salary.
Cumberland Infirmary, Carlisle. House Surgeon.—£70 per annum, and pupils' fee, with board, etc.
Cumberland Infirmary, Carlisle. Assistant House Surgeon.—£40 per annum, with board, etc.
Halifax Infirmary and Dispensary. Assistant House Surgeon.—£50 per annum, with board, etc.
London Temperance Hospital, Hampstead Road, N.W. Senior House Surgeon.—Fifty guineas per annum, with board, etc.
Morpeth Dispensary. House Surgeon.—£120 per annum, with furnished house, coals, and gas.
Queen's Hospital, Birmingham. Resident Physician.—£50 per annum, with board, etc.

**Non-resident Medical Officers.**

City of London Hospital for Diseases of the Chest, Victoria Park, E.—Assistant Physician. Candidates must be F. or M.R.C.P., Lond.
Norfolk and Norwich Hospital. Assistant Surgeon.

MEDICAL science, says a contemporary, was practically unknown a century ago ; but it is surprising what absurd superstitions were commonly believed. The eighteenth century had its "well-established system of copious and everlasting bleeding," like Holloway's pills, a cure for every disease. But according to Mr. G. Everitt, in his interesting book on "Doctors and Doctors," still more extraordinary cures were recommended in the old medical treatises. A great medical man of the seventeenth century gives the following "excellent cure for gout": "Take a young puppy, all one colour, if you can get such a one, and cut him in two pieces through the back, alive, and lay one side hot to the grieved place—the inner side I mean." The poor puppies must have had a bad time of it if the doctor's advice was generally carried out. This is what the same authority recommends for "squinancy" or quinsey : "Take a silk thread dipped in the blood of a mouse, and let the party swallow it down that is troubled with the squinancy, pain, or swelling in the throat, and it will cure him."

MR. C. S. LOCH, the Secretary of the Charity Organisation Society, writing to the *Pall Mall Gazette*, says: "In a note in your issue of Saturday last you ask for some further particulars respecting the estimate I made of the amount spent annually in charitable relief in London. In my evidence before the Select Committee, I said that, so far as I could judge, the sum was under three millions. I have since checked the figures, and set them out in the accompanying table. The total amounts to £2,457,695. I have included the medical charities (£655,380) among 'voluntary institutions.' I have excluded apprenticeship charities, and those classed as educational. To draw a satisfactory line between classes of charities to be included and those to be excluded is hardly possible, I think, with the data at present available. Thus, it might be better to include apprenticeship charities, but it is not possible to separate them throughout from the educational charities, and I have therefore excluded them together with the latter. Similarly, with a fuller knowledge of the details, one might decide to include under relief some educational charities, and yet to exclude others, such as 'exhibitions,' etc. At best, however, only a rough estimate can be framed. I have referred in the table to the chief sources of information, and given the general grounds for the estimates. About one or two of these there may be some divergence of opinion. To the estimate of charitable relief might be added the 'total relief of the poor' in London, as returned on p. 280 (Appendix E) of the Report of the Local Government Board for 1886-7 on 'Poor Rate: Receipts and Expenditure, 1885-6.' This amounts to £2,258,029. The total spent in relief would then amount to £4,715,724, exclusive of the sums noted in the table below, for which not even an approximate estimate can, in my opinion, be made."

AT the meeting of the Select Committee of the House of Lords on Poor Law Relief, held on Monday, Lord Kimberley presiding, the Rev. J. W. Horsley said he objected to the scheme of distinct schools for workhouse children, and considered such schools expensive. He would secure kindness in the boarding-out system by a careful selection of foster-parents. Mrs. Charles, who has had many years' experience as a poor-law guardian at Paddington, also recommended the boarding-out system, and thought it a mistake to send girls to district schools. The Rev. B. Waugh spoke strongly against the prevalent system of insuring children's lives.

THE North-West London Hospital, of which the ninth report is now published, seems to be rapidly extending its borders. A few years ago it was a children's hospital. It now provides 18 beds for children and 29 for adults—in all 47 : so that it may fairly claim to be a general hospital. It is placed in a district which, but for its presence, would be almost destitute of hospital accommodation. During the year the Baron De Hirsch contributed a liberal donation of £500, in memory of the Baron Lucien De Hirsch. Since the publication of the last report, one special bed has been added, in memory of Annette Mary Maud Cassel. The bed has been permanently endowed by Mr. E. Cassel by a donation of £500. In connexion with the hospital is a soup kitchen, which appears to owe its maintenance to the treasurer. This institution is said to have proved of great service to many of the poorer patients during the recent winter, no fewer than eighteen hundred persons having received a weekly supply of bread and soup. An earnest appeal is made for additional funds for carrying on the various good works already in hand and for enlarging the field of operations.

THE fourteenth annual report of the Hospital Saturday Fund has just been issued, containing a complete list of subscriptions for 1887. Last year the subscriptions amounted to a total of £11,187, out of which the sum of £10,000 was voted to 78 hospitals, 39 dispensaries, 3 surgical appliance committees, and 16 convalescent homes and other institutions. Several institutions appear in the list for the first time; among them is Guy's Hospital, which received the sum of £390. Amongst the items may be noted that of the cabmen's collections, £162, while £35 was taken on the river steamboats, and £425 was received from the Dean of Westminster, being a moiety of the proceeds of the great repetition service at the Westminster Abbey, in commemoration of the Queen's Jubilee. The total out-door collections slightly exceeded £4,500, and the workshop collection amounted to nearly £6,000. Attention is called to the fact that the fund is largely supplemented by local collections for local charities, so that the nominal amount of between £11,000 and £12,000 does not fairly represent the contributions of working men toward the hospitals and dispensaries of London. The Council, in referring to this matter, says : "This system of local parades and demonstrations for local institutions, while commendable as a means of relieving the local institutions, has an undoubted tendency to weaken the central organisation ; it should not be forgotten that one of the chief objects of this movement is to secure for working men a voice in the management of the hospitals and dispensary. So long as men are content to form a number of small independent bodies, they will be powerless for the purposes of hospital reform. If this fact is properly recognised, the London workman will see that his aim should be the establishment of one great central organisation, which, for this purpose, would exert great influence."

